# Contemporary dental student professionalism: moving towards a macro-level perspective

**DOI:** 10.1038/s41415-024-7297-8

**Published:** 2024-04-26

**Authors:** Isabelle M. Cunningham, Mark Gormley, Patricia Neville

**Affiliations:** 4141586665001 Senior Clinical Lecturer, Bristol Dental School, University of Bristol, UK; 4141586665002 Consultant Senior Lecturer in Oral Surgery, Bristol Dental School, University of Bristol, UK; 4141586665003 Senior Lecturer, Bristol Dental School, University of Bristol, UK

## Abstract

Defining professionalism and developing educational interventions that foster and assess student professionalism are integral to dental education. Nevertheless, conceptual, methodological and pedagogic differences define the academic field, leaving students, educators and the profession itself struggling to make meaningful progress on how best to elicit and monitor dental student professionalism. This article proposes that more progress can be made on this important issue when a contextualised, sociological assessment of dentistry and dental professionalism is undertaken. We contend that identifying some of the socio-cultural demands in UK dental students' lives, and acknowledging how these pressures shape their interactions with the UK dental education system, provides a nuanced and contemporaneous understanding of what it means to be an oral health care professional at a time of social and health care upheaval. Dental educators can use this insight to work towards being more understanding of and responsive to dental student professional development.

## Key points

- Most models of dental student professionalism adopt an individualist approach to professionalism, constructing it as a function of individual traits, attitudes, behaviours and knowledge.
- Missing from this academic discourse is recognition of the fact that dental students are also social, political, economic and cultural agents who routinely interact with patients, educational and health care providers, and professional regulators. This demanding context plays an equally important formative impact on professional development.
- Taking a more contextualised and sociological approach to professionalism allows us to better appreciate the unique characteristics of and challenges associated with being a dental professional in the twenty-first century. Dental educators can use this insight to work towards being more understanding of and responsive to dental student professional development.

## Introduction

In common with other health care professions, dentistry has been engaged in the task of defining professionalism and developing educational interventions that foster and assess student professionalism. This ‘professionalism project'^1^ shows no sign of slowing down. Despite a vast body of literature, there is no universally accepted definition of professionalism, nor is there shared understanding of best pedagogic practice for teaching and assessing it.^2^^,^^3^^,^^4^^,^^5^^,^^6^^,^^7^ In the absence of a standardised approach, it falls to educators to try and make sense of the breadth of ever-expanding literature and to grapple with the ‘greyness' of the definitions in order to attempt to explain the concept to students. We are not the first to articulate the challenges inherent in dentistry's professionalism project and we acknowledge the work conducted by countless scholars on this topic. However, in the face of student apathy and ambivalence towards professionalism teaching (despite attempts to develop innovative pedagogy) and dental educator frustration with student professionalism lapses, it is becoming increasingly evident to us that we need to look at this issue from a fresh perspective. We need to acknowledge the limits of dental professionalism academic discourse and, instead of seeing these limits as discussion endpoints, take them as opportunities to reframe what we mean by dental professionalism. This article is not intended to summarise the professionalism literature or conversations that have taken place so far. The reader is instead referred to earlier articles within the *British Dental Journal* which has positioned itself at the heart of the debate,^2^^,^^3^^,^^8^^,^^9^^,^^10^ particularly that of Hanks *et al.,*^2^ which summarises the professionalism conversation to date. Our aim is to build on these previous contributions by reinforcing why traditional perspectives on professionalism are outdated for UK dental education and professional practice, and to highlight the current direction of travel within the wider health care professionalism literature. We then discuss organisational, educational, societal and historical contextual factors which have typically received less attention in the literature but which are becoming increasingly relevant to professionalism in contemporary education. By also highlighting aspects of professionalism that are unique to dentistry, this article offers a view of dental professionalism that embraces a sociological and contextualised understanding of it.

## Moving from a nostalgic to a complexity view of professionalism

Until recently, health care professionalism was principally understood and defined by individual attributes, that is, values, attitudes and behaviours. Examples include altruism, trustworthiness, reliability and patient communication.^11^^,^^12^^,^^13^ Hafferty and Castellani^11^ identified the shortcomings of this model for medical professionalism, calling out its over-reliance on individual motives and behaviours as outdated and ‘nostalgic'. The health care professions are increasingly accepting that viewing professionalism as a set of individual characteristics is overly simplistic and reducing it to a set of learning outcomes devoid of context is problematic for educators.^2^^,^^14^ Contextual factors that are increasingly impacting on UK health care provision include: the complexities of providing efficient, cost-effective health care systems; the potentially harmful (iatrogenic) nature of health care;^15^ the rise in litigation; and the intractable nature of health inequalities.^16^ These macro-level factors remind us of the ‘wicked problem' that is health care provision.^17^ Linked to this, a patient safety movement emerged in the 1990s, foregrounding the principles of candour, accountability and transparency,^18^^,^^19^ plus a patient-centred care movement which advocates for providing care that respects and responds to the needs, preferences and values of the individual.^20^ Such movements have transformed health care provision and help us to re-appraise the clinician-patient encounter as an interplay of clinical knowledge and competencies with non-clinical skills, attitudes and behaviours, such as patient respect, teamworking, effective communication (with health care team, the patient and their family), accountability and reflective practice.^20^ As a result, professionalism within health care is increasingly being recognised as a dynamic, transactional process, one that is continually evolving and being shaped by structural considerations, as well as attentiveness to patient-specific needs and concerns.

To provide a description of professionalism within dentistry to ‘anchor' our discussion paper, we offer a 2013 definition from the dental literature, as cited in the 2020 General Dental Council (GDC) commissioned mixed method professionalism study.^21^ Professionalism is ‘the manner in which one reflects on and reconciles different aspects of professional practice, which demonstrates acceptance of professional responsibility and accountability. It is manifested in the manner in which work is carried out. The balance of the various aspects will vary with and be appropriate to the context while accountability means that the professional is expected to be able to justify his/her actions to the patient, the profession, the society in which they work and themselves'.^4^

It is evident that professionalism within the health care professions, including dentistry, is increasingly being understood as a complex and multifaceted construct. Linked to this, professional development is now being seen and studied as a dynamic, socially constructed process that involves the formation of professional identity,^22^^,^^23^ and both professionalism and its development are becoming accepted as inherently context dependent. Professional attributes do exist but they are subject to negotiation across different contexts.^1^^,^^13^^,^^24^ Not wishing to underplay the role of individual and inter-personal factors,^24^ we call for a contemporary dental professionalism model that is reframed from a macro-perspective, that is, one that takes into consideration the broader contextual influences that shape and determine today's dental students and educators, and how they engage with the discourse and practice of professionalism. Hafferty and Levinson argue that professionalism discourse must ‘move its current focus as a discourse that stresses individual motives and behaviours to one that includes a more macro-perspective on how systems and structures affect individuals and how organisations themselves might embody professional principles'.^1^

A contemporary understanding of professionalism requires interrogation through contextual lenses which focus on organisational, educational, societal and historical factors.

## The organisational context: the dental school and undergraduate programme

Accepting that there are parallels in educational and clinical contexts across the health care professions, we contend that dental education is unique in context, and that this uniqueness must be recognised and accounted for when considering a fresh perspective on dental professionalism. As an organisation, the dental school is well recognised as being a stressful learning environment.^25^^,^^26^ Although both medical and dental students undergo intense five-year curricula, the latter are required to carry out operative procedures on patients from the first or second year of study. They are supervised by qualified staff and procedure complexity is tailored to each year of training, but, unlike their medical counterparts (in the event they undertake invasive procedures), the luxury of direct one-to-one supervision is not routine, with group supervision being the norm. Despite negotiating unfamiliar and challenging operative procedures, dental students need to work safely, undertake independent real-time decision-making, and constantly manage their patients' needs. They are also expected to interact professionally with their patients and a diverse clinical team. It is true that medical students, nurses and allied health professionals also learn in authentic workplace settings and need to build professional relationships;^23^^,^^27^^,^^28^^,^^29^however, dental students assume ownership of patients from an early stage of training. This requires them to source and complete a sufficient range of procedures in order to demonstrate competence before graduation.^30^ Within the dental school setting, the scope of procedural skills that students encounter has increased over recent years, not least due to technological advances in biomaterials and operative techniques compared to the relatively narrow range of procedures (primarily amalgam and a small number of tooth-coloured materials) that students encountered around 30 years ago.^31^ The demand this places on contemporary undergraduates to become professionals who are discerning, up-to-date and evidence-based with all treatment modalities should not be underestimated. Finally, as a profession which requires fine motor skills, dentistry has a tendency to attract perfectionist traits.^32^ Coupled with the stressful programme, this can lead to the potential for mental health challenges.^33^^,^^34^ In summary, our students learn within a particularly challenging environment and shoulder a unique set of professional responsibilities and expectations. This calls into question the extent to which findings and opinions from the wider health care professionalism literature, which originate from different teaching contexts, can or should be applied to dental student professionalism.

## The organisational context: the dental profession

The dental profession is a specific organisation into which our new graduates enter. It is one which has changed immeasurably and continues to evolve. Most students will enter general practice and, although the pressures of NHS dentistry and running a business are longstanding ones, the growing financial pressures, contractual demands and litigation concerns affecting dental professionals is well-recognised.^8^^,^^21^^,^^35^ This has been acknowledged by the GDC in its recent review of undergraduate learning outcomes: *The safe practitioner*.^36^ This document rightly highlights the need for today's dental professionals to have greater awareness of contextual factors that impact on daily practice, to view patient complaints as an inevitable part of practice, to understand their own strengths and limitations, and to monitor and manage their mental health.

The dental profession has traditionally been dominated by biomedical and chairside approaches to oral health, whereas more upstream health measures, the social determinants of health, and health advocacy are arguably better embedded in medicine.^37^ However, there is now an appreciable shift from operative to preventive dentistry and a greater appreciation of social factors that influence oral health, as evidenced by the proposed changes to the UK dental contract,^38^ and the emphasis on prevention, health advocacy and patient communication in *The* *safe practitioner.*^36^ Unfortunately, despite this overdue shift in emphasis, most dental professionals in today's busy NHS and post-COVID practices have insufficient time to get to know their patients, with appointment time pressures risking professional-patient relationships and patient trust.^39^ Compounding this is the current chronic shortage and fast turnover of personnel within practices and corporates, in contrast to previous generations of dentists, many of who spent entire careers in well-established family practices.

## The wider educational context: contemporary higher education and health care

Today's students are likely to have high expectations from their university education, viewing the opportunity to obtain a degree as a right, rather than something which is earned. There is a growing body of higher education (HE) literature centred around unrealistic student expectations, as defined by the terms ‘student entitlement' and ‘student as customer'.^40^^,^^41^^,^^42^^,^^43^^,^^44^^,^^45^^,^^46^ This has been driven by rising university fees, the marketisation of HE and competition between institutions.^47^ Most HE establishments also face increasing resource pressures, with an inevitable impact on what they can realistically offer students. Tomlinson cautions that ‘as HE is reconfigured from a largely public good to one that serves largely private interests and values, a dominant ethic of rights and entitlement has entered the fray'.^46^ This is a particular concern for professional health care programmes which uphold the public good in the form of patient care. Universities are also increasingly expected to co-create learning experiences with students^48^ and to regularly respond to their feedback, as evidenced by the rise in institutional and national student evaluation schemes.^49^ There can be no doubt that the teacher-student power balance has shifted away from the teacher-dominated hierarchy traditionally seen within health care institutions,^50^ with the potential for students to challenge hierarchy and, in return, there is also the potential for teachers to question their professionalism. Within health care institutions where professionalism takes centre stage, the interactions and power dynamics between students and teachers is of particular relevance. Although there is discourse in the medical literature around issues such as the use of professional titles,^51^ and evidence that the increasing power of students can impact negatively on medical teachers,^52^ the complex interplays and tensions between dental students and their teachers requires further investigation. Despite a lack of evidence, we caution that dental teachers should consider the potential pitfalls of maintaining steep hierarchies. Gordon^51^ suggests that if we want students, staff or patients to speak up, then respect is more important than reverence. Interestingly, the NHS #hellomynameis campaign (which emphasises that appropriate introductions are part of compassionate care) advises that the use of names, rather than titles, is key to instilling a sense of belonging, flattening the hierarchy and improving patient safety.^53^ With respect to dress code for health care professionals, it is well-accepted that infection control and personal protective equipment requirements will dictate the parameters, but opinions on professional dress code, both clinical and non-clinical, within modern, diverse health care institutions, vary between staff and students. However, such workforce diversity is not often reflected in changes to institutional or regulator policy.^54^

## The societal context: today's student generation

Staff and students from multiple generations interact on a daily basis within dental schools. There is currently popular discussion and debate about the ‘generational gap' between students and their educators,^55^^,^^56^^,^^57^ and today's young adults are defined by terms such as Millennials, Generation Z (Gen Z) and Generation Me.^58^^,^^59^ While we acknowledge that the concept of generational gap has not yet emerged as a formal educational theory, there is evidence to suggest that our students have different lifestyles, learning styles, teaching expectations and career aspirations compared to previous generations. The majority of today's dental students are Gen Z^60^ and have had access to digital technologies and the internet since birth. Accepting that digital skill does not necessarily translate into digital literacy,^61^ our students are undoubtably better connected, with the majority embracing digital teaching methods and assuming instant information.^62^ While Gen Z are less likely to smoke and consume alcohol, a rise in the use of illegal substances poses a significant threat to their professionalism and fitness to practise.^63^ The decline in alcohol intake has in part been facilitated by social media, with concerns around ‘recorded regret' or worry about posts being uploaded while people are under the influence.^64^ Similarly, access to illegal substances has been made easier by social media and encrypted communication technologies. Many other potential professionalism pitfalls exist through the misuse of social media, such as the increasing prevalence of ‘sexting',^65^ breaches in patient confidentiality from posting clinical information without specific consent, or the use of inappropriate and inflammatory language online.^66^^,^^67^ Although GDC guidance reminds students that social media can blur the boundaries between public and private life, and that online behaviour can impact on professional image,^68^ there is evidence of ‘push back' from dental students against institutional regulation of their online activity,^69^ with some students feeling that a certain level of unprofessional behaviour is acceptable while at university, given they are still students. A recent mixed-methods GDC study supports this perspective to some extent, with the public appearing less concerned about non-working-life behaviours, instead focusing on a dentist's ability to deliver a good service.^21^ This contrasts with research from medicine, suggesting that the public may have higher expectations from doctors.^70^

Traditional professional behaviour is often taught as ethical, ‘religious-like' principles and has been argued that the concept of ‘good religious behaviour' aligns with socially acceptable or professional behaviour.^71^ However, Gen Z report declining religious affiliation, particularly within developed nations, and are the most likely group in history to identify as atheist or agnostic.^72^ It has therefore been suggested that medical professionals in western countries no longer have a focus on altruism^71^ and some experts are concerned that there is a narcissistic or inflated ‘sense of self' in both the Millennial and Gen Z cohorts.^59^ This has been associated with high grade expectation, lack of respect for faculty staff and poor acceptance of failure and feedback.^73^ That said, current students appear to have greater agency and voice than previous generations, being more likely to engage in social activism around important issues, such as equality, decolonisation or climate change. This suggests younger generations are more likely to challenge established norms and behaviours than their predecessors.^74^ One example of their collective agency is perhaps the current generation calling for a healthier work-life balance and protecting physical and mental health.^75^ It has been proposed that today's dental students are less resilient due to risk avoidance within a ‘safety culture' society.^76^ Older generations may use disparaging terms such as ‘the snowflake generation', but true strength may lie in highlighting those issues which have long been ignored, in expecting a more balanced dental career, safer working practices and avoiding burnout.^77^ To this end, many dental students are now considering a portfolio career by adding variety through activities such as teaching, developing a specialist interest, or working outside the profession.^78^ There may be professional challenges for dental students who have been raised in a society and education system which upholds agency and voice, but who are then subject to being ‘moulded' into professionals who are required to meet the expectations of health care teachers from earlier generations. Teachers from earlier generations are more likely to expect patients' interests to come first at all costs - a potential conflict with the search for better work-life balance by today's young professionals.

While current students appear less likely to conform, they are under greater pressure to perform. Admission into UK dental school remains highly competitive, with most requiring a minimum of three A grades at A-level, usually alongside strong GCSE grades and University Clinical Aptitude Test scores and voluntary work experience.^79^ A series of multiple mini interviews and/or aptitude or situational judgement tests usually follows this initial screening process, not only to ensure candidates are academically able, but also that they have the traits required for the profession.^80^ Preparation for this intensive entry process often starts from an early age and, in itself, may be the reason why our current students are less likely to have part-time jobs as teenagers, or to socialise frequently. The impact of COVID-19 lockdowns on the social experiences of our most recent undergraduate recruits must also be mentioned. Whatever the reason, reduced social opportunities inevitably alter student life experience compared with previous generations, which in turn may influence their professional perspectives and interactions with patients and staff. In summary, the emerging evidence for a societal ‘generational gap' should encourage dental educators to consider the lifestyles, learning styles and career preferences of today's students as part of learner-centred teaching.^81^ It is also a useful metaphor for educators to share their frustrations, challenges and successes around teaching and professionalism and could help to build stronger and educationally meaningful relationships with our students.^55^^,^^57^

## The societal context: public perspectives and expectations

The public perception of dentistry and dental professionalism is receiving greater attention, but it should not be assumed that perceptions mirror those of other health care professions. The nature of the dentist-patient relationship is a unique and complex one. Dental procedures and consultations involve the invasion of personal space and the dental environment is experienced by many as threatening and anxiety-provoking. This has been associated with a heightened dentist-patient power imbalance, patient passivity, paternalistic models of communication and lack of patient trust.^82^^,^^83^^,^^84^^,^^85^^,^^86^ Public expectations of the dental profession are also unique and changing. Within our contemporary consumerist society, and with the rise of social media, appearance is fast becoming the number one priority for many patients, rather than valuing oral health, as defined by the World Health Organisation.^87^^,^^88^^,^^89^ The dental profession is adapting to this with a rise in private cosmetic procedures.^90^ It could be argued that it is reasonable for dental professionalism discourse to place relatively less emphasis on altruism and servitude to society compared to other professions, such as medicine and nursing. Even aspiring students have been shown to factor in pragmatic considerations when selecting dentistry as a career, such as sociable working hours and a reliable salary, with less emphasis placed on vocational, caring aspects than nursing and medical students.^91^

## The historical context: decolonising dental professionalism

One of the achievements of the social movements #BlackLivesMatter and Decolonising the Curriculum has been their critical analysis of the knowledge systems upon which academic disciplines, including dentistry, are historically based. They highlight that a Western, Eurocentric/Anglo-Saxon/American worldview dominates academic knowledge construction, with ‘white men's' research being championed as valid knowledge and non-Western or indigenous health cultural systems and research being actively dismissed. The calls to diversify and decolonise dentistry^92^^,^^93^^,^^94^present a particular challenge to constructions of professionalism because they call out the extent to which dentistry predicates its understanding of professionalism on a historically specific, Western-orientated cultures framework.^95^ Dental educators' understanding of what constitutes professional and unprofessional behaviour is typically rooted in a particular construction of the archetypical ‘professional' who possesses an exemplar list of traits and values - impartial yet empathic, self-confident and self-assured, self-sacrificing, works long hours, with a clear sense of right and wrong and an ability to navigate ethical dilemmas with relative ease. This character and virtue-based representation of professionalism has been critiqued as being patriarchal, middle class and Victorian in nature.^96^^,^^97^^,^^98^ By adhering to this definition of professionalism, we are endorsing a model that is fundamentally ‘Western, white, heteronormative and male'.^7^ When presented in this way, it becomes evident that such a model is unrealistic: it ignores the existence of non-Western health care and belief systems, and makes non-Western dental student values and behaviours vulnerable to being classed as ‘unprofessional', even though they are accepted practice in their cultures.^99^ For example, some Eastern cultures are noted for being patriarchal, family- or collective-orientated, and the consenting process is understood as a collective one, with eldest sons being involved in health decisions being made about an older mother or relative.^100^^.^ Our new graduates must be well-prepared for the local societal context within which they will be practising, and for most, that will be increasingly multi-cultural. They should also be effective world citizens in today's global society.

## Conclusion

Dental professionals, both practitioners and educators, need to accept that traditional models of professionalism, with their over-emphasis on individual attributes, are outdated, and acknowledge that professionalism and professional development are complex and context-dependent constructs. We have argued that the discourse of dental professionalism needs a change of mindset. It requires, and deserves, an entirely fresh perspective, one that recognises the ‘macro context' and embraces the organisational, educational, societal and historical contexts that are shaping contemporary professionalism and educational practice. In particular, we must acknowledge that, and understand why, today's generation of students are different, and ensure this message reaches all those involved in dental education. A fresh perspective must also recognise that there are specific contexts which are unique to dental education and the profession, and recognise the cultural biases and assumptions that are inherent in professionalism discourse. We maintain that re-appraising dental professionalism along the contextual lines of enquiry we have identified will lead to a more nuanced and prescient understanding of the academic and pedagogic issues associated with dental professionalism education and better equip us to prepare today's graduates for contemporary professional practice. We look forward to the ongoing discussions which we hope this article initiates, and we aim to provide research findings that utilise this perspective to develop a new and beneficial professionalism model for dental educators.
